# The Clinicopathological Spectrum of Parathyroid Carcinoma

**DOI:** 10.3389/fendo.2019.00731

**Published:** 2019-10-23

**Authors:** Amit Akirov, Sylvia L. Asa, Vincent Larouche, Ozgur Mete, Anna M. Sawka, Raymond Jang, Shereen Ezzat

**Affiliations:** ^1^Department of Endocrine Oncology, Princess Margaret Cancer Centre, Toronto, ON, Canada; ^2^Institute of Endocrinology, Beilinson Hospital, Petach Tikva, Israel; ^3^Sackler School of Medicine, Tel Aviv University, Tel Aviv, Israel; ^4^Department of Pathology, University Health Network, University of Toronto, Toronto, ON, Canada; ^5^Division of Endocrinology and Metabolism, Department of Medicine, Jewish General Hospital, McGill University, Montreal, QC, Canada; ^6^Division of Endocrinology, University Health Network and University of Toronto, Toronto, ON, Canada; ^7^Department of Medicine, Princess Margaret Cancer Centre, University of Toronto, Toronto, ON, Canada

**Keywords:** hyperparathyroidism, parathyroid carcinoma, thyroid nodule, parathyroidectomy, parathyroid disease, Endocrine Oncology

## Abstract

**Background:** Parathyroid carcinoma is rare, representing <1% of primary hyperparathyroidism cases.

**Methods:** Retrospective data of patients referred for evaluation of parathyroid disease between 2001 and 2018 were reviewed. The goal was to describe the clinical presentation, histopathologic characteristics, and treatment outcomes of parathyroid carcinoma.

**Results:** We identified 8 cases of parathyroid carcinoma from the outpatient practice of a quaternary care Endocrine Oncology practice in Toronto, Canada. The clinical presentation was as follows: 5/8 cases (62.5%) of symptomatic hypercalcemia and 3/8 cases (37.5%) of a suspicious thyroid nodule. Hypercalcemia was evident in all 7 cases with pre-operative calcium measurements. Histopathologic features included: vascular invasion in 7/8 cases (87.5%) and immunohistochemical loss of either parafibromin, retinoblastoma, or p27 in all 8 cases. Additional treatment included: external beam radiotherapy in 5/8 cases (62.5%), chemotherapy for 2/8 patients (25%), and additional surgery for 3/8 patients (37.5%). Only 2 patients (25%) had long-term remission following surgical treatment, and the others had either persistent (3 patients) or recurrent disease (3 patients). Five patients developed metastatic disease, all involving lung. In one of two patients treated with Sorafenib there was evidence of regression of lung metastases. One patient died of disease progression.

**Conclusion:** In this series of patients with parathyroid carcinoma largely presenting with symptomatic hypercalcemia and angioinvasive disease, only a minority achieved a durable remission. Lung was the most common site of distant metastasis. Surgery led to remission in two cases, but none of the six patients with persistent or recurrent disease ultimately achieved disease remission.

## Introduction

The diagnosis of parathyroid carcinoma is usually determined at pathology examination following surgery for a suspected parathyroid neoplasm or for another indication, as there are no reliable preoperative tests to confirm this diagnosis (1). While most patients with parathyroid cancer present with functioning lesions and hyperparathyroidism, some may have normal parathyroid hormone (PTH) levels (2, 3). As this is a very rare disease, accounting for <1% of all cases of primary hyperparathyroidism, data regarding the diagnosis, treatment modalities and outcomes are limited (1, 4, 5).

The diagnosis of parathyroid carcinoma must be made by the pathologist on the surgically resected tumor and is, therefore, typically post-operative. Occasionally there can be preoperative documentation of distant or lymph node metastasis or gross local invasion into adjacent organs, but these are more often identified by the pathologist; the diagnosis may also be confirmed in the absence of gross invasion or metastasis when there is unequivocal perineural, lymphatic, and/or vascular invasion identified on histopathology. The findings of fibrosis, necrosis and increased mitotic activity are worrisome histological findings but not do not necessarily warrant the diagnosis of malignancy, as they can be seen in parathyroid hyperplasia or adenomas that have been physically manipulated (6–9).

The American Joint Committee on Cancer (AJCC) eighth edition of cancer staging has recently proposed a classification of these tumors. Staging of the primary tumor (T) is as follows: localized to the parathyroid gland with limited extension to soft tissue (T1); direct invasion into the thyroid gland (T2); direct invasion into recurrent laryngeal nerve, esophagus, trachea, skeletal muscle, adjacent lymph nodes, or thymus (T3); or direct invasion into major blood vessel or spine (T4). Staging of regional lymph nodes (N) includes: no regional lymph node metastasis (N0); metastasis to regional lymph nodes (N1), including metastasis to level VI or VII (N1a) or to levels I, II, III, IV, V, or retropharyngeal nodes (N1b). Staging of distant metastasis (M) is, as for other tumors: no distant metastasis (N0) or with evidence of distant metastasis (M1). However, as the available data on tumor characteristics and prognosis are limited, no prognostic stage groups were suggested (10).

Previous studies have shown that surgical intervention has the best chance of disease control and long-term remission (4, 5). Sandelin et al. reported a median time from initial surgery to first recurrence of 33 months (range 1–228 months), with median survival time from the first recurrence to death of 28 months (range 0–129 months). The 5 year and the 10 year overall survival in their study was 85 and 70%, respectively. A review from the National Cancer Database reported a 5-year survival rate of 86% and 10-year survival rate of 49% (11).

There is some uncertainty regarding the benefit of external beam radiotherapy (EBRT) for prevention of recurrent disease. The American Association of Endocrine Surgeons published guidelines for definitive management of primary hyperparathyroidism stating that adjuvant EBRT should not be routinely performed after surgical resection of parathyroid cancer, suggesting that radiation should be reserved as a palliative option (12). There are very limited data in the medical literature regarding other treatment modalities specifically targeted against this rare endocrine carcinoma. Limited guidelines are available for medical treatment of parathyroid cancer, and the aforementioned guidelines by the American Association of Endocrine Surgeons did not include specific recommendations for drug treatment.

The aim of this study was to describe the clinical presentation, histopathologic characteristics, and treatment outcomes of patients with surgically-treated parathyroid carcinoma.

## Methods

In this retrospective chart review, we reviewed the records of all patients referred for evaluation and management of parathyroid disease at the Endocrine Oncology Clinic of one of the authors (SE) at the Princess Margaret Cancer Centre, Toronto, Ontario, between 2001 and 2018. Cases were identified using clinical scheduling records which were linked to billing diagnostic codes, and screening of the electronic and paper medical records of those with relevant billing codes.

The data from the electronic or paper health records was abstracted and entered into an electronic spreadsheet (Excel, Microsoft) by a study investigator (AA) and checked for accuracy by the treating physician (SE). The diagnosis of parathyroid cancer was based on the presence of invasive histopathologic features (e.g., vascular invasion, lymphatic invasion, perineural invasion or local gross malignant invasion into surrounding structures) of the primary tumor or the presence of biopsy-proven distant and/or nodal metastatic disease.

The study was restricted to surgically treated cases, where review of the surgical pathology was performed by the same two experienced endocrine pathologists (SLA; OM) to ensure consistency of detailed histopathologic features. All the pathology samples were reviewed and their reports in the electronic medical record detailed the status of vascular invasion (defined by tumor cells invading through a vessel wall and/or intravascular tumor cells admixed with thrombus), lymphatic invasion, perineural invasion, invasion of other tissues, surgical margin involvement, immunohistochemical reactivity of p27, parafibromin, and Rb, as well as the Ki67 labeling index. Both pathologists agreed in advance on the criteria for the diagnosis of vascular invasion (13). All patients were followed by a single Endocrine Oncologist (SE). The typical post-operative follow-up protocol included clinical, biochemical, and radiological follow-up. Remission was defined by the presence of low or appropriate PTH and calcium levels and no evidence of structural disease on cross-sectional imaging.

We summarized the data descriptively, including numbers and percentages for categorical data and mean or median and standard deviation (SD) or range for continuous data. The study was approved by the institutional research ethics board of University Health Network. Informed consent was not required for this retrospective chart review.

## Results

We reviewed 219 patients referred for parathyroid disease; 175 patients were found to have hyperparathyroidism, 14 patients were managed for hypoparathyroidism, and 30 patients were found to have normal parathyroid function. Of the 175 patients with hyperparathyroidism, 149 individuals had primary hyperparathyroidism, including 9 patients with parathyroid carcinoma; 22 cases were secondary hyperparathyroidism; 3 were tertiary hyperparathyroidism; one patient had familial hypocalciuric hypercalcemia. Of the parathyroid cancer cases, one was excluded as the pathology sample from another institution was not available for review. We ultimately included 8 patients (5 women, 3 men) with parathyroid carcinoma meeting our study eligibility criteria (with mean age ± SD at diagnosis of 53.5 ± 10.4 years, Table 1). The mean ± SD follow-up was 6.2 ± 3.8 years. The family history was negative for parathyroid carcinoma in all cases.

**Table 1:** Clinical data and therapeutic approaches for eight patients with parathyroid carcinoma.

**Patient number**	**Follow-up (years)**	**Stage**	**Surgical intervention**	**Radiation (Gy)**	**Chemotherapy**	**Metastasis at baseline**	**Metastasis during FU**	**Outcome**	**Exitus**
#1	3.5	T3N0M1	Total Thyroidectomy	56	–	Lung, hip	Lung, hip liver	Persistent disease	–
#2	6.4	T1NxM0	Rt. Hemithyroidecomy	–	–	–	–	Remission	–
#3	3.6	T2N1M0	Total Thyroidectomy	60	–	–	lung	Persistent disease	+ (Parathyroid cancer)
#4	4.6	T1N0M0	Right Subtotal Thyroidectomy + Right Parathyroidectomy + Mediastinal Dissection + Right neck dissection	–	–	–	–	Remission	–
#5	6.7	T1NxM0	Three Surgeries – Resection of right superior PTA >> Right subtotal thyroidectomy + removal of multiple small nodules in the infero-lateral aspect of the gland and sternomastoid muscle >> Right central neck dissection	66	–	–	–	Initial remission, followed by recurrent disease.	–
#6	13.9	T2NxM0	Two Surgeries – Right Hemithyroidectomy + Right Inferior Parathyroidectomy >> Right neck dissection	50	–	–	lung	Initial remission, followed by recurrent disease.	+ (Colon adenocarcinoma)
#7	2	T2N1M0	Total Thyroidectomy + Central and Left neck dissection	–	SORAFENIB	–	lung	Persistent disease	–
#8	8.8	T1NxM0	Lt. Parathyroidectomy + Small margin of the thyroid >> Thoracotomy with neck dissection, resection of mediastinal mass, part of the manubrium and clavicular head, sternum, first rib	66	CAP-TEM >> Etoposide-Cisplatin >> investigational therapy >> Sorafenib >> Everolimus	–	liver, skeletal, lung, peritoneum, lymph nodes.	Initial remission, followed by recurrent disease	–

### Clinical Presentation

The diagnosis of parathyroid carcinoma was made at the time of histopathologic examination after surgical resection in all cases. Calcium levels were elevated in all seven patients who had these measured preoperatively (mean ± SD before surgery 3.7 ± 0.8 mmol/L; mean PTH ± SD levels before surgery 64.9 ± 63.3 pmol/L). Pre-operative management was not standardized, as some of these patients were referred only after the histopathologic diagnosis of parathyroid carcinoma was made after surgery.

Investigation of a neck mass led to diagnosis of a thyroid nodule in three patients (patients #1–3) who were referred for thyroidectomy; the pathology report following surgery revealed parathyroid carcinoma. One of these patients (patient #3), had been investigated for weight loss and constipation, leading to the diagnosis of hyperparathyroidism, and a neck ultrasound revealed a 3.5 cm hypoechoic nodule in the lower pole of the right thyroid that was suspicious for differentiated thyroid cancer on fine-needle aspiration biopsy. The patient underwent total thyroidectomy and the pathology was consistent with angioinvasive and widely invasive parathyroid carcinoma arising from the right parathyroid gland and involving the thyroid and the painted resection margins. In five other patients (patients #4–8), surgery was indicated for primary hyperparathyroidism identified during investigation of symptomatic hypercalcemia, including one case of kidney stones (patient #6), hypercalcemia-induced pancreatitis (patient #5), and other symptoms associated with high calcium levels such as bony aches, polyuria, polydipsia, and constipation (patients #4, #7, and #8). One of these patients (patient #4) presented to the emergency room with severe life-threatening hypercalcemia (calcium 5.0 mmol/L, PTH 27.8 pmol/L) and mental changes, was hospitalized in the intensive care unit and an ultrasound examination revealed a 6 cm mass in the right paratracheal area, as well as a mass in her right ipsilateral thyroid. This patient had an emergent right subtotal thyroidectomy with right parathyroidectomy, mediastinal dissection and right zone 6 neck dissection. The pathology revealed parathyroid carcinoma, as well as follicular variant papillary thyroid carcinoma.

In this case series, the parathyroid tumor was found mainly on the right side (6/8 patients, 75%). Only one patient was diagnosed with distant metastases immediately following the diagnosis of parathyroid carcinoma (patient #1), and these included metastases to the lungs, bone (hip) and liver. Four other patients (patient #3, #6, #7, and #8) had no evidence of metastasis at baseline, but developed metastases during follow-up, with evidence of lung metastases in all of them. Germline DNA testing for *CDC73*/*HRPT2* was performed based on the documentation of loss of parafibromin by immunohistochemistry staining in three patients (patients #1, #7, #8); germline pathogenic mutations were not found in these patients.

All patients had a history of kidney stones, which were symptomatic in all but one patient (patient #4). There was no history of bony fractures in any of the patients, and preoperative bone mineral density results were not available. One patient (patient #8) had prior surgeries on his right humerus and left hip, all performed at another institution; these were possibly brown tumors.

### Histopathologic Findings

The pathology characteristics are summarized in Table 2. The mean tumor size was 49.0 ± 26.5 mm in the 6 patients for whom preoperative imaging results were available, as the remaining 2 patients were initially treated elsewhere. Vascular invasion was evident in 7 patients, and indeterminate in one patient (patient #1). Lymph node involvement was reported in two patients (patient #3 and #7); positive margins were evident in six patients (patients #1–4, #6, and #8). The adjacent thyroid was invaded in all of these patients; local gross invasion to other adjacent tissues was identified in one patient (patient #1) and perineural invasion was documented in one case (patient #7).

**Table 2:** Pathology features of 8 parathyroid carcinomas.

**Patient number**	**Age at diagnosis**	**Gender**	**Size (mm)**	**Vascular invasion**	**Lymph Node involvement**	**Positive margins**	**Invasion to adjacent tissue**	**Chromogranin**	**p27 Loss**	**Parafibromin loss**	**Rb loss**	**Necrosis**	**MIB1%**
#1	54	F	37	Indeterminate	0/5	+	Thyroid, Esophageal Margin	+	+	+	+	–	
#2	53	F	34	+		+	–	+	+	−	+	–	5%
#3	73	M	35	+	5-Jan	+	Thyroid	+	+	minimal loss	+	+	>20%
#4	51	F	65	+	0/2	+	–		+	–	–		5%
#5	41	F	NA	+	0	NA	–	+	+	+	+		4%
#6	54	F	NA	+		+	Thyroid	–	+	–	+	+	NA
#7	61	M	27	+	Jan-38	−	Thyroid	+	+	+	–	+	19.60%
#8	41	M	96	+		+	–			+	+		14.98%

In addition to immunohistochemical biomarkers of parathyroid differentiation including chromogranin-A and parathyroid hormone and GATA3, the expression profile of biomarkers that may represent potential markers of malignancy or targets for novel therapies was also examined in these cases. Loss of p27 was found in six cases (patients #1, #2, #4, #5–7), while in 4 cases there was parafibromin loss (patients #1, #5, #7, and #8). Loss of Rb was reported in six patients (patients #1–3, #5, #6, and #8). Staining for p53 was done in 7 of 8 cases and showed no evidence of misexpression in all seven (i.e., no complete loss and no diffuse positivity). In two cases examined for further ancillary testing, galectin-3 was positive in both cases (patients #4 and #7), while BCL2 staining was retained in one (patient #4) and reduced in the other (patient #7). The Ki67 labeling index was available for 6 of 8 tumors; in three patients it was approximately 5% (patients #2, #4, #5), while in three others it was ≥15% (patients #3, #7, and #8). Of note, there were five patients who developed metastases during follow-up; the Ki67 labeling index was available for three of these patients, and in all three it was ≥15% in the primary tumor. The Ki67 data of the other two patients with metastatic disease was not available.

The TNM staging of the cancer is shown in Table 1. One patient had metastatic disease (M1) on presentation, two patients had evidence of regional lymph node metastasis (N1), while in 4 patients the nodal status was unknown (Nx). Half of the patients had tumor localized to the parathyroid gland with limited extension to soft tissue (T1) (Table 1).

Pre-operative FNA was performed in two of the three patients (patients #1–3) who were referred for surgical intervention due to suspected thyroid nodules; these resulted in an indeterminate result in one (patient #2) and a diagnosis of “suspicious for papillary thyroid cancer” in the other (patient #3). All these patients were found to harbor parathyroid carcinoma, with no evidence of thyroid malignancy. However, in four of six patients with primary hyperparathyroidism (patients #4, #5, #7, and #8), who were referred for surgical intervention for parathyroid disease, there was an incidental finding of papillary thyroid cancer, with three cases of microcarcinoma (patients #5, #7, and #8), and one case (patient #4) diagnosed with a 1.8 cm oncocytic follicular variant papillary thyroid carcinoma.

### Treatment

As shown in Table 1, five patients underwent only one surgical intervention. Two patients had total thyroidectomy (patients #1 and #3), one patient (patient #2) had right hemithyroidecomy for a suspicious nodule. Two patients (patients #6 and #8) had two surgeries (patient #6: right hemithyroidectomy with right inferior parathyroidectomy followed by neck dissection; patient #8: subtotal thyroidectomy with left inferior parathyroidectomy, followed by thoracotomy with neck dissection, resection of mediastinal mass, part of the manubrium and clavicular head, sternum, and first rib), while another patient (patient #5) had three surgeries, including initial resection of the right superior parathyroid gland, followed by right subtotal thyroidectomy with removal of multiple small nodules in the infero-lateral aspect of the gland and sternomastoid muscle, and eventually a right central neck dissection.

Regarding the two patients without a diagnosis of hyperparathyroidism prior to the surgery (patients #1 and #2), one of them (patient #1) did not have PTH levels measured before surgery, however during post-operative follow up she was diagnosed with hyperparathyroidism and hypercalcemia. The other patient (patient #2) developed hypocalcemia with hungry bone syndrome after surgery, although she only had right hemithyroidectomy, as the indication for surgery was a suspected thyroid lesion. These findings, along with the fact that all patients had a history of kidney stones, suggest that all the patients in this cohort had functional hyperparathyroidism likely attributable to their disease.

External beam radiation treatment (EBRT) to the neck was administered to five patients (patients #1, #3, #5, #6, and #8), at doses ranging between 50 and 66 Gy. This line of treatment was chosen due to persistent disease following surgery in 4 of 5 patients (patients #1, #3, #5, and #8). Right recurrent laryngeal palsy before the surgery and uncontrolled hypercalcemia following the parathyroid surgery was the indication for EBRT in the remaining patient (patient #6). Three patients went into remission following surgical and radiation treatment (patients #5, #6, and #8), but two of them (patients #6, and #8) were later diagnosed with recurrent local and distant disease. In the other two patients (patients #1, and #3) there was evidence of residual disease, both local and distant, and one (patient #3) passed away from his parathyroid cancer during the follow-up period.

Additional systemic therapies were used to control hypercalcemia; cinacalcet was used in one patient (patient #6), zoledronate was used in two patients (patients #7 and #8) but later was switched to denosumab 120 mg, which was also used in another patient (patient #3) with good response.

Sorafenib was used in two patients (patient #7 and #8) with evidence of metastatic disease following parathyroid surgery. Patient #7 received initially denosumab 120 mg every month for hypercalcemia, but as he developed lung metastasis, treatment with sorafenib 400 mg, twice daily, was initiated and imaging after 3 months revealed regression of the lung nodules. Patient #8 began treatment with sorafenib following disease progression with previous treatments, including combination of capecitabine and temozolomide, cisplatin and etoposide, zoledronate, denosumab, and an investigational drug. Due to disease progression the patient was then placed on everolimus.

### Outcomes

#### Remission

Remission was defined by the presence of low or appropriate PTH and calcium levels and no evidence of structural disease on cross-sectional imaging.

Following the first surgical intervention, five patients went into remission (patients #2, #4, #5, #6, and #8); including one patient (patient #6) that shortly after right hemithyroidectomy and right inferior parathyroidectomy had EBRT.

Two of the patients (patients #2 and #4) remained in remission at the end of follow-up, with no further treatment and without evidence of recurrent biochemical or structural disease. Both of them developed hypocalcemia after the surgery requiring treatment with calcium and calcitriol, although in both cases, the surgical intervention did not include removal of all parathyroid glands or total thyroidectomy. In both patients with sustained remission, pathology did not reveal lymph node involvement, and Ki67 was 5% in both cases.

In both patients that went into long-term remission at the end of follow-up after parathyroid surgery, the initial tumor was localized to the parathyroid gland with limited extension to soft tissue (T1), with no clear evidence of regional lymph node metastasis (N0 or Nx).

#### Recurrent Disease

Three patients achieved remission after the first surgery, but later developed recurrent disease (patients #5, #6, and #8). The time interval between the first surgery and the recurrence was 22, 36, and 58 months, respectively.

In all the patients with evidence of recurrent disease, pathology indicated vascular invasion, and Rb loss. Loss of parafibromin and p27 loss were evident in 2/3 patients. Two of the patients with recurrent disease developed distant metastasis during follow-up (patients #6 and #8). All three patients had another surgery for their recurrent disease, and while one patient (patient #6) had already received EBRT post-operatively, the other two patients were treated with EBRT for their recurrent disease. However, EBRT treatment did not lead to remission, and all three patients were left with residual disease.

*Patient #5* was in remission for 22 months following parathyroidectomy, but reappearance of symptomatic hypercalcemia and elevated PTH levels led to the detection of recurrent disease. She was referred for neck exploration and right subtotal thyroidectomy with removal of multiple small nodules in the infero-lateral aspect of the gland and sternomastoid muscle. Surgical pathology revealed recurrent parathyroid carcinoma, as well as a focus of papillary thyroid microcarcinoma. Following this second surgery, her PTH levels did not normalize and she still had hypercalcemia. Imaging studies showed paratracheal nodules on the right and 1 year later she was referred for right central neck dissection, where pathology once again confirmed parathyroid carcinoma. The combination of this surgical intervention with postoperative EBRT normalized her PTH levels and at last follow-up there was no evidence of structural disease on imaging of the head and neck, other than two tiny non-specific nodules in the right thyroid bed.

*Patient #6* was diagnosed with recurrent parathyroid carcinoma <3 years following her initial surgery. She had recurrent structural and biochemical disease, with evidence of hypercalcemia and elevated PTH levels, as well as right recurrent laryngeal nerve palsy and lung metastasis. The patient was started on Cinacalcet for hypercalcemia and underwent a right neck dissection; pathology confirmed recurrent parathyroid carcinoma. The patient was later diagnosed with colon adenocarcinoma that was treated with surgery and chemotherapy but ultimately led to her demise.

*Patient #8* did not receive EBRT following his first surgery, and he was well and free of disease for almost 4 years. Prior to his recurrent parathyroid carcinoma, he was found to have a large liver mass which was biopsied and reported to be hepatocellular carcinoma. Three years later, he developed a growing neck mass, involving the mediastinum and left neck, which on core biopsy was found to be parathyroid carcinoma. The patient had extensive surgery, including thoracotomy with neck dissection, resection of mediastinal mass, part of the manubrium and clavicular head, sternum, and the first rib. Unfortunately, the tumor was adherent to the thoracic inlet, and although it was dissected off the trachea and lateral wall of the esophagus, disease was left *in situ*. Pathology again revealed angioinvasive parathyroid carcinoma and the patient completed post-operative EBRT. His PTH levels normalized and he did not require treatment for hypercalcemia, however, several months later, a new liver lesion was found, and this time a core biopsy revealed metastatic parathyroid carcinoma, which was not amenable to surgical excision. For that reason, he was started on a neuroendocrine tumor (NET)-type of chemotherapy regimen in the form of capecitabine and temozolomide. After 2 cycles there was progression of his liver disease that continued despite an additional two cycles of chemotherapy. The patient was switched to combination of cisplatin and etoposide, and given that the disease outside of his liver was relatively modest in burden, he also underwent bland liver embolization. Following this combination treatment, imaging studies showed widespread skeletal metastases, that were treated with EBRT to the focal bone lesions, in addition to zoledronate, which was later replaced by monthly denosumab 120 mg. Due to further disease progression, with evidence of metastases in the liver, skeleton, and lung, the patient was enrolled in a phase 1 trial to which he did not respond, as he manifested progression in the form of new peritoneal metastases. He was referred for peptide receptor radiotherapy (PRRT) but was found to be ineligible due to minimal uptake on Ga68-dotatate PET imaging. The patient was subsequently started on sorafenib but continued to progress and recently was switched to everolimus.

In all three patients with initial remission that were later found to have recurrent disease, the initial tumor was either localized to the parathyroid gland with limited extension to soft tissue (T1, patients #5 and #8) or with direct invasion into the thyroid gland (patient #6), and the lymph node status was unknown (Nx). However all three exhibited angioinvasion.

#### Persistent Disease

Three patients (patients #1, #3, and #7) had residual biochemical and structural disease following their first surgical intervention that persisted to the end of follow-up. In all three patients there was evidence of metastases, either at diagnosis (patient #1), or during follow-up (patients #3 and #7). In all these patients, pathology indicated invasion to adjacent tissue, which involved the thyroid gland in all cases, but only patient #1 had esophageal involvement. Loss of p27 was evident in all three, The Ki67 labeling index was available for two of the patients (patient #3 and #7) and was high (>20%, and 19.60%, respectively).

Three other patients (patient #5, #6, and #8) had recurrent disease, that was treated (as described above) but all of them were left with persistent disease following their recurrence.

*Patient #1* was referred for total thyroidectomy for a suspicious thyroid nodule, which turned out to be parathyroid carcinoma. This was followed shortly after total thyroidectomy by EBRT with a reduction of PTH levels from nine- to five-fold the upper limit of normal and normal calcium levels. At diagnosis, he had evidence of metastasis in the lungs, hip, and liver. He had radiation treatment directed to his hip, followed by prosthesis insertion.

*Patient #3* was suspected to have thyroid cancer and primary hyperparathyroidism and underwent total thyroidectomy; pathology identified an angioinvasive and widely invasive parathyroid carcinoma. Following surgery, his PTH remained elevated, but calcium levels were in the normal range. He had EBRT to his neck. Although there was no evidence of metastasis at baseline, the patient developed lung metastasis during follow-up as well as recurrent hypercalcemia. Zoledronic acid failed to control hypercalcemia, followed by monthly denosumab 120 mg with good biochemical control. The patient had slowly progressing, low-volume disease, with enlarging pulmonary metastases, then manifested progression in the neck with a paraesophageal mass invading the esophageal wall. He was started on everolimus but could not tolerate the treatment at a dosage of 10 mg daily, which was subsequently reduced to 5 mg but this was discontinued as it proved ineffective. He was later started on sorafenib 400 mg daily, but this was discontinued after a few months due to severe walking difficulties and drop foot. The patient died of this disease 6 years after his parathyroid carcinoma diagnosis.

*Patient #7* was diagnosed with primary hyperparathyroidism, as previously reported (14), following investigation of symptomatic hypercalcemia, with diffuse bony aches, constipation, polyuria, polydipsia, and weight loss of more than 25 pounds. Imaging identified a left thyroid nodule and a left neck mass; biopsy of the left neck mass was suspicious for a neuroendocrine neoplasm. The patient had total thyroidectomy with central and left neck dissection. Pathology revealed an intrathyroidal 2.7 cm parathyroid carcinoma and metastatic carcinoma in 2 left neck lymph nodes, PTH decreased after the surgery but remained elevated, with hypercalcemia and hypophosphatemia. Imaging did not reveal any residual neck disease, but there were multiple pulmonary nodules, consistent with metastatic disease. The patient was started on denosumab 120 mg every month for his hypercalcemia. Later, the patient started treatment with sorafenib and imaging after 3 months showed regression of the lung nodules.

In all three patients with residual disease, the initial staging indicated evidence of distant metastasis (M1, patient #1), or regional lymph node involvement (N1, patients #3 and #7). In all three cases, the tumor was either invading the thyroid gland (T2, patients #3 and #7), or more extensively (T3, patient #1).

## Discussion

This case series confirms the finding in previous studies that the diagnosis of parathyroid carcinoma is typically established only by pathology examination after surgery (1). As expected, primary hyperparathyroidism was the most common indication for surgery (15); thyroid nodule was another important reason for further investigation and intervention, which eventually led to diagnosis of parathyroid cancer. Another interesting finding is that all patients had a history of kidney stones, including those without a pre-operative diagnosis of hyperparathyroidism, suggesting previous undiagnosed hyperparathyroidism. This claim is supported by the fact that the patients without a pre-surgical diagnosis of hyperparathyroidism developed either hypercalcemic hyperparathyroidism, or hungry bone syndrome after surgery, even if the procedure was only a hemithyroidectomy.

Parathyroid carcinoma is often difficult to diagnose preoperatively, thus potentially limiting the scope of surgical intervention. As long-term survival is largely dependent on the extent of the primary surgical resection, it is of great importance to consider parathyroid carcinoma in the differential diagnosis of hyperparathyroidism. While our series included patients with symptoms related to hyperparathyroidism and hypercalcemia, there is significant variability in clinical patterns of the disease, and others have described asymptomatic patients with parathyroid carcinoma without clinical or biochemical clues to the diagnosis (16).

The histopathology diagnosis of parathyroid carcinoma in all our patients was made on the basis of vascular invasion or invasion of adjacent structures such as esophagus in one case, as well as loss of parafibromin, Rb, or p27 expression (13). These biomarkers have been proposed to support a diagnosis of malignancy in borderline cases without clear-cut angioinvasion or documented metastases at presentation. Erovic et al. investigated the expression profile of potential immunohistochemical biomarkers of parathyroid cancer, and completed staining for 34 proteins involved in angiogenesis, inflammation, cell adhesion, cell cycle, and apoptosis. They reported that a panel that includes BCL-2a, parafibromin, Rb, and p27 may be helpful in the assessment of parathyroid neoplasms, but there are additional possible biomarkers that may be helpful, though the data are preliminary. Parafibromin, Rb, and p27 are involved in cell cycle, while BCL-2a is involved in apoptosis. A panel that includes BCL-2a, parafibromin, Rb, and p27 was shown to be very helpful in the assessment of atypical parathyroid neoplasms, when there was no evidence of angioinvasion, perineural invasion, or gross local invasion into adjacent organs or metastasis (13). These data supported previous studies focusing on the biomarkers for parathyroid carcinoma (17–19). Recently, reports described novel mutations in genes that mediate chromosome organization, DNA repair, and cell cycle, and occasional mutations in MAPK signaling and immune response (including *PTEN, NF1, KDR, PIK3CA*, and *TSC2*). Additionally, epigenetic studies have described changes in DNA methylation, histone modifications, microRNA dysregulation, and unusual circular RNAs (20–22). Kutahyalioglu et al. evaluated tumor-specific genetic changes using next-generation sequencing (NGS) panels in 11 patients with parathyroid carcinoma, reporting mutations identified in the PI3K (4/11 patients) and TP53 (3/11 patients) pathways. In addition, mutations were identified in genes that were not previously reported in parathyroid carcinoma, including *SDHA, TERT* promoter, and *DICER1*. Actionable mutations were found in more than half of the patients (23). While no longer routinely used, silver-stained nucleolar organizer region (AgNOR) analysis may serve as an additional tool for the histological evaluation of parathyroid lesions to distinguish adenomatous from cancerous ones, as higher AgNORs per nucleus (NORA) scores were noted in malignant cases (24).

Of note, invasion into the thyroid gland should not be considered a feature of malignancy, as parathyroid glands are frequently located within or immediately adjacent to thyroid tissue and benign parathyroid lesions can occur within the thyroid gland (25). This is a weakness in the new proposed AJCC staging system that identifies this feature as a criterion for upstaging a parathyroid carcinoma.

As stated previously, the first and foremost treatment for parathyroid carcinoma was surgical intervention (12). In two cases, the surgery that was performed for a different indication was the only treatment required for the parathyroid carcinoma, as the patients remained in remission at the end of follow-up. However, while the recommended treatment is en bloc resection (12), in one of the two patients in long-term remission, surgery was limited to hemithyroidectomy/parathyroidectomy, with no neck dissection.

EBRT was administered to five patients; all these patients had recurrent or persistent disease and there was evidence of distant metastasis in four of them, with lung involvement in all cases. Our results confirm previous reports that could not show prominent response to radiotherapy and thus did not recommend this as a routine treatment for parathyroid cancer (12, 26, 27). Erovic et al. reported 11 of 16 patients who underwent postoperative radiotherapy, and this was one of the largest series with respect to the use of adjuvant radiotherapy. Of these 11 patients, 7 developed recurrent disease, including 3 of 4 patients who had positive surgical margins (27). Lee et al. reported that <10% of their 224 patients with parathyroid cancer received radiation therapy, and this treatment was not associated with an improved survival rate (26).

Both patients in long-term remission went into remission immediately after their first surgery, and did not require any additional treatment until the end of follow-up, which was at least 4 years in these patients. The pathology examination of these patients revealed that none of these three patients had lymph node metastasis or loss of parafibromin expression, and Ki67 was in the low range (~5%).

A novel finding in our series is the high number of patients with additional other malignancies. In our cohort, four patients had thyroid cancer (diagnosed concurrently with the parathyroid cancer at thyroid surgery), one patient had colorectal cancer, and another had hepatocellular carcinoma. The diagnosis of incidental thyroid cancer is not surprising, given that it largely consisted of low risk subclinical disease. Campenni et al. completed a systematic literature search exploring the association between parathyroid cancer and thyroid disease, reporting 21 cases of parathyroid cancer with thyroid disease, including 10 cases of concomitant parathyroid carcinoma and thyroid cancer, mainly papillary thyroid carcinoma (28). In that series, the parathyroid cancer mean diameter was higher in those with both malignancies, and there was a slight predominance for the left side (28). Similarly, in our cohort, thyroid carcinoma was evident in 2 of 2 patients with parathyroid carcinoma on the left side, compared to 2 of 7 patients with parathyroid malignancy on the right side.

In the three patients with recurrent disease and those with persistent disease, further intervention, whether in the form of another surgery, EBRT, or chemotherapy, did not lead to clinical remission. Five of the six patients with persistent or recurrent disease developed metastases during the follow-up. As EBRT did not have a profound effect on tumor progression, chemotherapy was used in two patients. The lack of response to EBRT in these cases is in line with the recommendation by the American Association of Endocrine Surgeons that discourage the use of EBRT, other than for palliative reasons (12).One patient was treated with the tyrosine kinase inhibitor (TKI) sorafenib; imaging studies after several months showed regression of the lung metastases, but the second patient did not respond to several lines of chemotherapy, including sorafenib. The data in the literature indicate variable response to a wide range of chemotherapeutic regimens, including Dacarbazine, Cyclophosphamide, or Capecitabine, alone or in combination (4, 29). The response of one of our patients to Sorafenib, with regression of his pulmonary nodules, was reported previously in a case report (7). The response to Sorafenib, a multi-kinase inhibitor that blocks cell proliferation and angiogenesis, may stem from its effect against vascular endothelial growth factor-receptor and platelet-derived growth factor receptor, which may be highly expressed in parathyroid cancers (7, 15).

The recently defined AJCC staging based on the tumor characteristic at the time of initial presentation may aid in predicting the prognosis of these patients (10). In our case series, all patients with evidence of regional lymph node metastasis (N1) or distant metastasis (M1) had residual disease following parathyroid surgery and required additional treatment. On the other hand, all four patients with a tumor localized to the parathyroid gland with limited extension to soft tissue (T1) went into remission following the surgery, although two of them later developed recurrent disease and required further treatment.

The limitations of this study include the retrospective nature, small number of cases, and lack of a standardized pre-operative management, as some of these patients were referred only after the histopathologic diagnosis of parathyroid carcinoma was made after surgery and none of them had a clear preoperative suspicion of malignancy. In addition, as this study was performed at a single outpatient practice of a quaternary care Endocrine Oncology specialist practice, there is a potential for referral and selection bias. However, as parathyroid cancer is a very rare entity, with limited data available in the medical literature, this study may add important insights to the body of evidence. In addition, the long-term follow-up, treatment in a specialized center with involvement of a highly experienced team and various available treatment options, are among the strengths of this study.

In accordance with the guidelines for management of primary hyperparathyroidism by the American Association of Endocrine Surgeons, the diagnosis of parathyroid carcinoma should be considered in cases of primary hyperparathyroidism with marked elevation of PTH levels and severe hypercalcemia (12). Complete resection, which occasionally requires en bloc resection of adherent tissues is recommended as first line therapy. Genetic testing should be considered in patients with parathyroid carcinoma including those with parafibromin loss by immunohistochemistry even in the absence of family history or HPJT-related manifestations (30, 31). Adjuvant treatment, including repeat surgery, radiotherapy or medical treatment, should be considered on an individualized basis especially when remission is not achieved following surgery. Regular surveillance is recommended, including biochemical monitoring of PTH and calcium homeostasis, as well as imaging of the neck with neck ultrasound, parathyroid scan and/or head and neck computed tomography or magnetic resonance imaging, Initially, biochemical and radiographic monitoring is recommended every 3–6 months, depending on the aggressiveness of disease and the response to treatment. In case of long-term remission, the interval can be increased to once yearly.

In conclusion, our findings suggest that parathyroid cancer is usually identified only post-operatively, during the pathology examination, pointing to the importance of an experienced pathology team. While long-term remission is possible with surgery, in patients with evidence of residual disease after the intervention and in those with recurrent disease during follow-up, chances of cure are very low. Targeted therapies may prove to be an important treatment option for those patients with distant metastatic disease.

## Data Availability Statement

The datasets generated for this study are available on request to the corresponding author.

## Ethics Statement

The studies involving human participants were reviewed and approved by University Health Network. Written informed consent for participation was not required for this study in accordance with the national legislation and the institutional requirements.

## Author's Note

AA had full access to all the data in the study and takes responsibility for the integrity of the data and the accuracy of the data analysis. Data available on request from the authors.

## Author Contributions

AA, SA, VL, AS, and SE: substantial contributions to conception and design, acquisition of data or analysis and interpretation of data, drafting the article or revising it critically for important intellectual content, final approval of the version to be published. OM: acquisition of data or analysis and interpretation of data, drafting the article or revising it critically for important intellectual content, final approval of the version to be published. RJ: drafting the article or revising it critically for important intellectual content, final approval of the version to be published.

### Conflict of Interest

The authors declare that the research was conducted in the absence of any commercial or financial relationships that could be construed as a potential conflict of interest.

## References

[1] Dotzenrath C Goretzki PE Sarbia M Cupisti K Feldkamp J Röher HD. Parathyroid carcinoma: problems in diagnosis and the need for radical surgery even in recurrent disease. Eur J Surg Oncol. (2001) 27:383–9. 10.1053/ejso.2001.1122 11417985

[2] Giessler GA Beech DJ Nonfunctional parathyroid carcinoma Goetz. J Natl Med Assoc. (2001) 93:251–5. 11491274 PMC2594040

[3] Mazeh H Prus D Freund HR. Incidental non-functional parathyroid carcinoma identified during thyroidectomy. Isr Med Assoc J. (2008) 10:659. 18847176

[4] Fraker DL. Update on the management of parathyroid tumors. Curr Opin Oncol. (2000) 12:41–8. 10.1097/00001622-200001000-00007 10687728

[5] Sandelin K Auer G Bondeson L Grimelius L Farnebo LO. Prognostic factors in parathyroid cancer: a review of 95 cases. World J Surg. (1992) 16:724–31. 10.1007/BF02067369 1413841

[6] Kim J Horowitz G Hong M Orsini M Asa SL Higgins K. The dangers of parathyroid biopsy. J Otolaryngol Head Neck Surg. (2017) 46:2–5. 10.1186/s40463-016-0178-7 28061891 PMC5219743

[7] Rozhinskaya L Pigarova E Sabanova E Mamedova E Voronkova I Krupinova J. Diagnosis and treatment challenges of parathyroid carcinoma in a 27-year-old woman with multiple lung metastases. Endocrinol Diabetes Metab Case Rep. (2017) 2017:16–0113. 10.1530/EDM-16-0113 28458892 PMC5404464

[8] LLoyd R Osamura R Kloppel G Rosai J WHO Classification of Tumours of Endocrine Organs. 4th edn Lyon: IARC Press (2017).

[9] Duan K Mete Ö. Parathyroid carcinoma: Diagnosis and clinical implications. Turk Patoloji Derg. (2015) 31:80–97. 10.5146/tjpath.2015.01316 26177319

[10] Landry C Wang T Asare E Parathyroid. In: Amin M, editor. AJCC Cancer Staging Manual. 8th edn New York, NY: Springer International Publishing (2017). p. 903.

[11] Hundahl SA. Two hundred eighty-six cases of parathyroid carcinoma treated in the U.S. between 1985-1995: a National Cancer Data Base Report. The American College of Surgeons Commission on Cancer and the American Cancer Society. Cancer. (1999) 86:538–44. 10430265 10.1002/(sici)1097-0142(19990801)86:3<538::aid-cncr25>3.0.co;2-k

[12] Wilhelm S Wang T Ruan D Lee J Asa S Duh Q. The American Association of endocrine surgeons guidelines for definitive management of primary hyperparathyroidism. JAMA Surg. (2016) 151:959–68. 10.1001/jamasurg.2016.2310 27532368

[13] Erovic BM Harris L Jamali M Goldstein DP Irish JC Asa SL. Biomarkers of parathyroid carcinoma. Endocr Pathol. (2012) 23:221–31. 10.1007/s12022-012-9222-y 23001705

[14] Alharbi N Asa SL Szybowska M Kim RH Ezzat S. Intrathyroidal parathyroid carcinoma: an atypical thyroid lesion. Front Endocrinol. (2018) 9:641. 10.3389/fendo.2018.00641 30455668 PMC6230986

[15] Schaapveld M Jorna FH Aben KKH Haak HR Plukker JTM Links TP. Incidence and prognosis of parathyroid gland carcinoma: a population-based study in the Netherlands estimating the preoperative diagnosis. Am J Surg. (2011) 202:590–7. 10.1016/j.amjsurg.2010.09.025 21861982

[16] Campennì A Ruggeri RM Sindoni A Giovinazzo S Calbo L Ieni A. Parathyroid carcinoma as a challenging diagnosis: report of three cases. Hormones. (2012) 11:368–76. 10.14310/horm.2002.1367 22908071

[17] Cryns VL Rubio MP Thor AD Louis DN Arnold A. p53 abnromalities in in human carcinoma. J Clin Endocrinol Metab. (1994) 78:1320–4. 10.1210/jcem.78.6.8200932 8200932

[18] Cryns VL Thor A Xu HJ Hu SX Wierman ME Vickery AL. Loss of the retinoblastoma tumor-suppressor gene in parathyroid carcinoma. N Engl J Med. (1994) 330:757–61. 10.1056/NEJM199403173301105 7906387

[19] Arnold A. Genetic basis of endocrine disease 5. Molecular genetics of parathyroid gland neoplasia. J Intern Med. (1993) 77:574–83. 10.1210/jcem.77.5.8077300 8077300

[20] Clarke CN Katsonis P Hsu TK Koire AM Silva-Figueroa A Christakis I. Comprehensive genomic characterization of parathyroid cancer identifies novel candidate driver mutations and core pathways. J Endocr Soc. (2019) 3:544–59. 10.1210/js.2018-00043 30788456 PMC6372985

[21] Cardoso L Stevenson M Thakker RV. Molecular genetics of syndromic and non-syndromic forms of parathyroid carcinoma. Hum Mutat. (2017) 38:1621–48. 10.1002/humu.23337 28881068 PMC5698716

[22] Hu Y Zhang X Cui M Wang M Su Z Liao Q Zhao Y. Circular RNA profile of parathyroid neoplasms: analysis of co-expression networks of circular RNAs and mRNAs. RNA Biol. (2019) 16:1228–36. 10.1080/15476286.2019.1622962 31213128 PMC6693544

[23] Kutahyalioglu M Nguyen HT Kwatampora L Clarke C Silva A Ibrahim E. Genetic profiling as a clinical tool in advanced parathyroid carcinoma. J Cancer Res Clin Oncol. (2019) 145:1977–86. 10.1007/s00432-019-02945-9 31309300 PMC11810207

[24] Tuccari G Abbona GC Giuffrè G Papotti M Gasparri G Barresi G. AgNOR quantity as a prognostic tool in hyperplastic and neoplastic parathyroid glands. Virchows Arch. (2000) 437:298–303. 10.1007/s004280000228 11037351

[25] Asa SL Mete O Parathyroids. In: Mills SE, editor. Histology for Pathologists. 5th ed Philadelphia, PA: Wolters Kluwer (2019). p. 1201–24.

[26] Lee PK Jarosek SL Virnig BA Evasovich M Tuttle TM. Trends in the incidence and treatment of parathyroid cancer in the United States. Cancer. (2007) 109:1736–41. 10.1002/cncr.22599 17372919

[27] Erovic B Goldstein D Kim D Mete O Brierley J Tsang R. Parathyroid cancer: outcome analysis of 16 patients treated at the Princess Margaret Hospital. Head Neck. (2013) 35:35–9. 10.1002/hed.22908 22290780

[28] Campennì A Giovinazzo S Pignata SA Di Mauro F Santoro D Curtò L. Association of parathyroid carcinoma and thyroid disorders: a clinical review. Endocrine. (2017) 56:19–26. 10.1007/s12020-016-1147-7 27744598

[29] Okamoto T Iihara M Obara T Tsukada T. Parathyroid carcinoma: Etiology, diagnosis, and treatment. World J Surg. (2009) 33:2343–54. 10.1007/s00268-009-9999-0 19350316

[30] Gill AJ Lim G Cheung VKY Andrici J Perry-Keene JL Paik J. Parafibromin-deficient (HPT-JT Type, CDC73 Mutated) parathyroid tumors demonstrate distinctive morphologic features. Am J Surg Pathol. (2019) 43:35–46. 10.1097/PAS.0000000000001017 29324469 PMC6296846

[31] Van Der Tuin K Tops CMJ Adank MA Cobben JM Hamdy NAT Jongmans MC. CDC73-related disorders: Clinical manifestations and case detection in primary hyperparathyroidism. J Clin Endocrinol Metab. (2017) 102:4534–40. 10.1210/jc.2017-01249 29040582

